# Phenotypic Characterization of LEA Rat: A New Rat Model of Nonobese Type 2 Diabetes

**DOI:** 10.1155/2013/986462

**Published:** 2013-02-26

**Authors:** Tadashi Okamura, Xiang Yuan Pei, Ichiro Miyoshi, Yukiko Shimizu, Rieko Takanashi-Yanobu, Yasumasa Mototani, Takao Kanai, Jo Satoh, Noriko Kimura, Noriyuki Kasai

**Affiliations:** ^1^Department of Laboratory Animal Medicine, National Center for Global Health and Medicine, Tokyo 162-8655, Japan; ^2^Department of Infectious Diseases, National Center for Global Health and Medicine, Tokyo 162-8655, Japan; ^3^Institute for Animal Experimentation, Tohoku University Graduate School of Medicine, Sendai 980-8575, Japan; ^4^Department of Comparative and Experimental Medicine, Nagoya City University Graduate School of Medical Sciences, Nagoya 467-8601, Japan; ^5^Center for Experimental Animal Science, Nagoya City University Graduate School of Medical Sciences, Nagoya 467-8601, Japan; ^6^Institute of Laboratory Animals, Tokyo Women's Medical University, Tokyo 162-8666, Japan; ^7^Division of Diabetes and Metabolism, Department of Internal Medicine, Iwate Medical University School of Medicine, Morioka 020-8505, Japan; ^8^Pathology Section, Department of Clinical Research, National Hospital Organization Hakodate National Hospital, Hakodate 041-8512, Japan

## Abstract

Animal models have provided important information for the genetics and pathophysiology of diabetes. Here we have established a novel, nonobese rat strain with spontaneous diabetes, Long-Evans Agouti (LEA) rat derived from Long-Evans (LE) strain. The incidence of diabetes in the males was 10% at 6 months of age and 86% at 14 months, while none of the females developed diabetes. The blood glucose level in LEA male rats was between 200 and 300 mg/dl at 120 min according to OGTT. The glucose intolerance in correspondence with the impairment of insulin secretion was observed in male rats, which was the main cause of diabetes in LEA rats. Histological examination revealed that the reduction of *β*-cell mass was caused by progressive fibrosis in pancreatic islets in age-dependent manner. The intracytoplasmic hyaline droplet accumulation and the disappearance of tubular epithelial cell layer associated with thickening of basement membrane were evident in renal proximal tubules. The body mass index and glycaemic response to exogenous insulin were comparable to those of control rats. The unique characteristics of LEA rat are a great advantage not only to analyze the progression of diabetes, but also to disclose the genes involved in type 2 diabetes mellitus.

## 1. Introduction

 Diabetes mellitus is a heterogeneous group of metabolic diseases that is characterised by hyperglycaemia. It can result in blindness, kidney and heart disease, stroke, loss of limbs, and reduced life expectancy if left untreated and is recognized as a primary threat to human health in the 21st century [1]. Type 2 diabetes mellitus is the most common form of diabetes and accounts for approximately 90% of cases; its development is controlled by interactions between multiple genetic and environmental factors [2–4]. The genetics and pathophysiology of type 2 diabetes remain poorly understood because detailed investigations, such as genetic dissection, have been restricted in humans for practical and ethical reasons. Animal models of type 2 diabetes mellitus have provided important information, and many rat strains of spontaneous diabetes have been reported [5–11]. Among these strains, Goto-Kakizaki (GK), Otsuka Long-Evans Tokushima Fatty (OLETF), and Spontaneously Diabetic Torii (SDT) rats have been widely used for elucidating the genes responsible for the development of diabetes, the physiological course of the disease, and the complications related to diabetes [12–14]. The different diabetes model strains exhibit different aspects of the disease, and thus additional animal models are needed to elucidate the complete pathogenesis of diabetes.

We have developed a novel, nonobese rat strain with spontaneous diabetes, Long-Evans Agouti (LEA) rat, which was established from a Long-Evans closed colony together with the Long-Evans Cinnamon (LEC) rat [15]. LEA rats spontaneously develop hyperglycaemia and glucosuria. They do not exhibit any signs of obesity throughout their lives but experience late onset of the disease and exhibit histological changes (macrophage infiltration and fibrosis) of the pancreatic islets. Thus, LEA rats may serve as a new model of nonobese type 2 diabetes mellitus caused by impairing insulin secretion. In the present study, we examined the pathophysiological characteristics of the strain and demonstrated that the LEA rat is a useful model of human nonobese diabetes.

## 2. Materials and Methods

### 2.1. Animals

 LEA rats, also known as LEA/SENDAI or SENDAI rats, were maintained at the Institute for Animal Experimentation, Tohoku University Graduate School of Medicine, Japan. Wistar rats were purchased from Japan SLC (Hamamatsu, Japan) and were used as controls. All rats were housed in air-conditioned animal rooms at an ambient temperature of 23 ± 3°C and relative humidity of 50 ± 10%, under specific pathogen-free conditions with a 12 h light/dark cycle. Food, consisting of a Labo MR standard diet (Nosan, Yokohama, Japan), and water were available *ad libitum*. The fat content of the diet was 4.1%. All animal care procedures were approved by the Animal Care and Use Committee of Tohoku University Graduate School of Medicine, and complied with the procedures of the *Guide for the Care and Use of Laboratory Animals of Tohoku University*.

### 2.2. Examination of Clinical Features

 Urinary glucose and protein were monitored at 1-month intervals using Uro-paper AG2 (Eiken, Tokyo, Japan). The detection limits of the Uro-paper were 10–20 mg/dL for urine protein and 40–60 mg/dL for urine glucose. The body weight (BW) and body length (BL), that is, the distance from the nose to the anus, of five male rats (6 months old) were measured. The body mass index (BMI) was calculated as BMI=BW(g)/BL(cm)^2^. 

### 2.3. Oral Glucose Tolerance Test (OGTT)

Glucose tolerance was estimated by the oral glucose tolerance test (OGTT). After 16 h fast, a dose of 2 g/kg BW of glucose was given orally, and blood samples were collected from the tail vein at 0, 30, 60, 90, and 120 min after loading. The blood glucose levels were measured with a Glutest EII blood glucose monitoring meter with a monitoring range of 40–500 mg/dL (Sanwa Kagaku, Nagoya, Japan). The rats were classified according to the three-grade system of diabetes mellitus (DM), impaired glucose tolerance (IGT), and normal. DM was defined as 120 min blood glucose levels of ≥200 mg/dL. IGT was defined as 120 min blood glucose levels between 140 and 199 mg/dL. Neither DM nor IGT was defined as normal.

### 2.4. Plasma Insulin Concentration and Insulin Tolerance Test

 The plasma insulin concentration was determined as immunoreactive insulin (IRI) by ELISA with a rat insulin assay kit (Morinaga Milk Industry, Yokohama, Japan) using separated plasma from the blood collected from the tail vein at 0, 30, 60, 90, and 120 min after glucose loading. To study early-phase insulin secretion, an OGTT was performed on male rats (2 and 12 months of age) as described previously, and blood glucose levels (BG) and insulin concentrations (IRI) at 0 and 30 min after glucose loading were measured. The insulinogenic index (ΔI.I.) was calculated as follows: ΔI.I. = ΔIRI/ΔBG, where ΔIRI and ΔBG are the differences between their respective values at 0 and 30 min. An insulin tolerance test was performed in 12-month-old, nonfasting male rats. The animals were intraperitoneally challenged with a dose of 0.75 U/kg BW of human insulin (Novolin R; Novo Nordisk, Denmark). Blood samples were drawn from the tail vein at different time points, and glucose levels were determined as described previously. 

### 2.5. Histological Analyses

 The tissues from rats were fixed overnight at 4°C in phosphate-buffered saline (PBS) that contained 4% paraformaldehyde. They were rinsed with PBS, dehydrated, embedded in paraffin, cut into 5-*μ*m-thick sections, and stained with haematoxylin and eosin (H&E). For insulin detection, the pancreatic sections were processed for immunostaining by an indirect method using Guinea pig anti-insulin polyclonal antibody (1 : 100, DACO, Carpinteria, CA) as the primary antibody and peroxidase-labelled goat anti-Guinea pig IgG antibody (1 : 200, Chemicon International, Temecula, CA) as the secondary antibody. The specific reactions were visualised with a DAB substrate kit (Vector, Burlingame, CA). To distinguish the inflammatory cells infiltrated in the islets, additional deparaffinised sections of pancreas were processed for immunostaining using mouse monoclonal antibodies against rat CD4 clone W3/25 (MCA 55R; Abd Serotec, Oxford, UK), CD8 clone OX-8 (MCA48R; AbD Serotec), CD45RA clone OX-33 (MCA340G, AbD Serotec), and macrophage antigen clone ED1 (MCA341, AbD Serotec). Specific reactions were visualised with a SAB-PO kit (Nichirei, Tokyo, Japan). Kidney sections were stained with Masson's Trichrome.

### 2.6. Examination of the *β*-Cell Volume Relative to the Pancreas Volume

 The volume of *β*-cells relative to the pancreas volume was calculated as the proportion of the total area of *β*-cells to the total area of pancreatic tissue, according to the method of Bouwens et al. [16], with some modifications. In brief, three serial paraffin sections (5-*μ*m-thick) of pancreatic tissue from three animals of each strain were obtained at intervals of 100 *μ*m. The sections were immunostained with Guinea pig anti-insulin antibodies (1 : 100) and analysed under an Olympus BX51 microscope (Olympus, Tokyo, Japan) connected to a computer running the WinROOF software (Mitani Corp., Tokyo, Japan). The image analysis quantified the total pancreatic tissue area and the insulin-positive area, permitting the calculation of the ratio of islet *β*-cell area to total pancreatic area. To ensure that any change in the relative *β*-cell volume was not attributable to a change in the size of individual *β*-cells, the density of nuclei per insulin-positive area was also measured in 20 islets of the five rats of each strain.

### 2.7. Statistical Analyses

 The results are expressed as means ± SD. Differences were analysed using the Student's *t*-test. A *P* value <0.05 was considered statistically significant.

## 3. Results

### 3.1. Incidence of Diabetes

**Figure 1:**
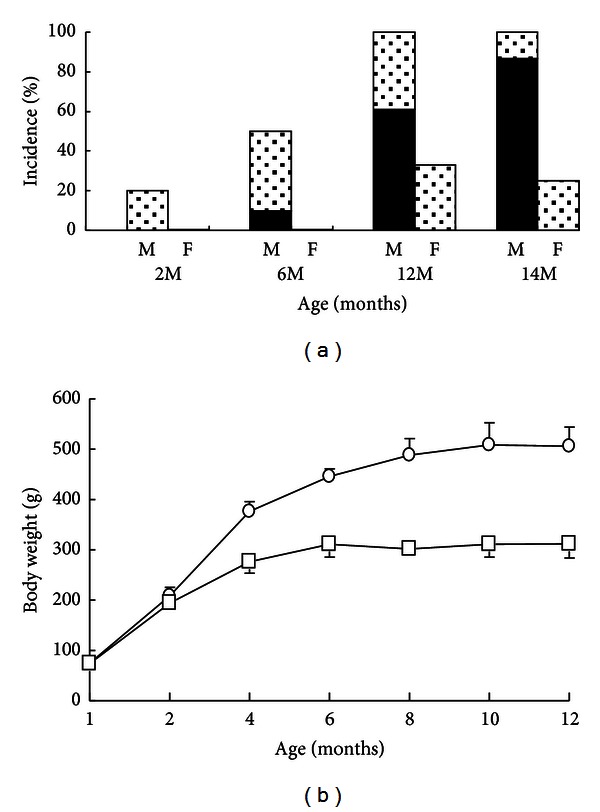
(a) Incidence of diabetes mellitus in LEA rats as determined by OGTT. Dotted and closed bars indicate IGT and DM, respectively. M and F indicate male and female, respectively. *n* = 32 for males at each age; *n* = 26 for females at each age. (b) Changes of body weight in male (open circle, *n* = 5) and female (open square, *n* = 5) LEA rats.

 The incidence of diabetes in LEA rats as determined by OGTT is shown in Figure 1(a). Diabetes mellitus was observed only in male rats, and its incidence increased with age: 10%, 61%, and 86% at 6, 12, and 14 months of age, respectively. IGT was observed at 2 months of age in the rats. The onset of diabetes was not observed in females, although 33% of the females showed only IGT at 12 months of age. As the onset of diabetes differed according to sex, only male rats were used in the experiments. Glucosuria appeared at 5 months of age, before the onset of diabetes in male rats, and was present in 100% of the males at 8 months of age. In female rats, glucosuria appeared at 7 months of age and was present in 100% of the females at 9 months (Table 1). Proteinuria appeared at 6 months of age, concomitant with the onset of diabetes, in male rats and was present in 57% of the males at 9 months of age, whereas proteinuria appeared in 20% of female rats at 9 months of age and did not exceed 30% of the females thereafter.

### 3.2. Body Weight and Survival Rate

 The average BWs of male and female LEA rat increased gradually throughout the experimental period and were 506 ± 37.8 g (*n* = 5) and 312 ± 27.7 g (*n* = 5) at the 12 months of age, respectively (Figure 1(b)). A significant decrease in BW could not be observed even after the onset of diabetes. The BMI of the LEA rats at 6 months of age (0.57 ± 0.02 g/cm^2^, *N* = 5) was not significantly different from that of the control Wistar rats (0.59 ± 0.02 g/cm^2^, *N* = 5), confirming that the LEA rats were nonobese. 

The survival rate of LEA rats was examined(Supplementary Figure 1 available online at http://dx.doi.org/10.1155/2013/986462). We found that 95% of the male rats survived to 12 months of age, and 50% survived to 22 months of age. The survival rate of male LEA rats was not significantly different from that of normal control Wistar rats, which indicates that diabetes does not influence the survival of LEA rats.

### 3.3. Glucose Tolerance and Insulin Response to Oral Glucose Loading

**Figure 2:**
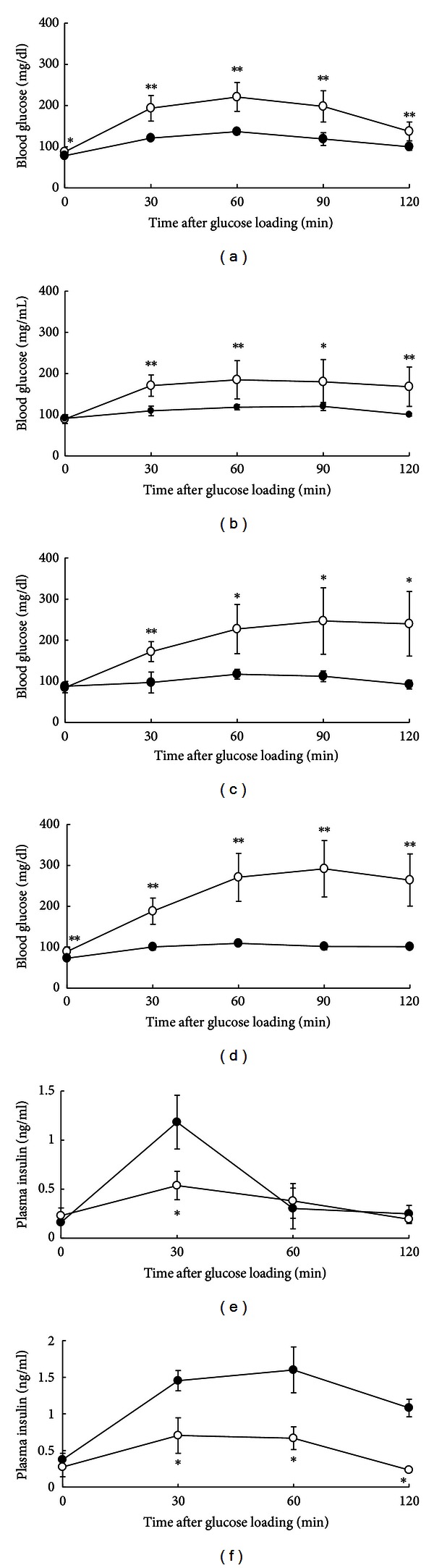
Blood glucose levels after glucose loading in male LEA (*n* = 11, open circle) and Wistar rats (*n* = 5, closed circle) at 2 months of age (a), in male LEA (*n* = 12, open circle) and male Wistar rats (*n* = 4, closed circle) at 6 months of age (b), in male LEA (*n* = 13, open circle) and Wistar rats (*n* = 3, closed circle) at 12 months of age (c), in male LEA (*n* = 15, open circle) and Wistar rats (*n* = 3, closed circle) at 14 months of age (d). Plasma insulin levels after glucose loading in male LEA (*n* = 3, open circle) and Wistar rats (*n* = 4, closed circle) at 2 months of age (e), in male LEA (*n* = 5, open circle) and Wistar rats (*n* = 3, closed circle) at 12 months of age (f). Each value is expressed as the mean ± SD. *P < 0.05. **P < 0.01.

 The results of the OGTT in male rats at different ages are shown in Figure 2. Two-month-old male LEA rats showed impaired glucose tolerance compared with age-matched male Wistar rats (Figure 2(a)). At 12 and 14 months of age, the LEA rats presented with typical diabetic glucose levels of ≥200 mg/dL at 120 min after glucose loading (Figures 2(c) and 2(d)). The Wistar rats did not show any change in blood glucose level in relation to age.

 The plasma insulin concentrations in male rats at 2 months of age showed that the pre-OGTT values did not differ among the rats, whereas the values at 30 min after glucose loading were significantly lower in LEA rats (Figure 2(e)). The plasma insulin level was significantly lower in LEA rats at 12 months of age after glucose loading (Figure 2(f)). These results indicate that LEA rats have impairment of insulin secretion in response to glucose stimulation. The low insulin levels measured at 30 min after glucose loading suggest that LEA rats have a decreased ability to secrete insulin at an early phase of the disease (Figures 2(e) and 2(f)). The insulinogenic index (ΔI.I.) of male LEA rats was significantly lower at both 2 and 12 months of age compared with that of Wistar rats (Table 2), which indicates that early-phase insulin secretion is significantly impaired at an early age. 

**Figure 3:**
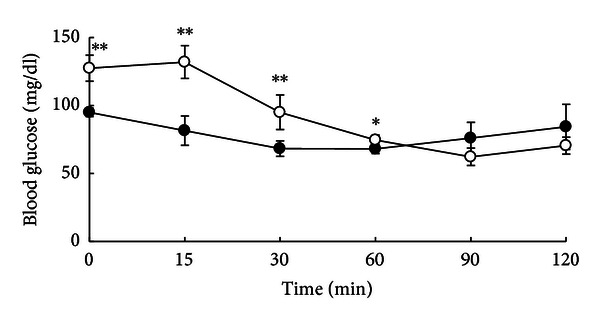
Insulin tolerance test. Blood glucose levels were measured after abdominal administration of insulin (0.75 IU/Kg) to nonfasting male LEA (*n* = 4, open circle) and Wistar rats (*n* = 4, closed circle) at 12 months of age. Significant reduction (*P* < 0.01) in blood glucose levels of LEA and Wistar rats was shown with measuring times after insulin injection. Each value is expressed as the mean ± SD. *P < 0.05. **P < 0.01.

 We performed an insulin tolerance test on nonfasting, 12-month-old male rats by intraperitoneal injection of insulin, to examine insulin response (Figure 3). Before insulin injection, the blood glucose levels were significantly higher in LEA rats than in Wistar rats. After insulin injection, the blood glucose levels in LEA rats significantly decreased by maximum 51.1% over 120 min to the same levels observed in the Wistar rats. These results indicate that LEA rats have a normal glycaemic response to exogenous insulin and are not insulin-resistant.

### 3.4. Pathological Changes of Pancreatic Islets in Male LEA Rat

**Figure 4:**
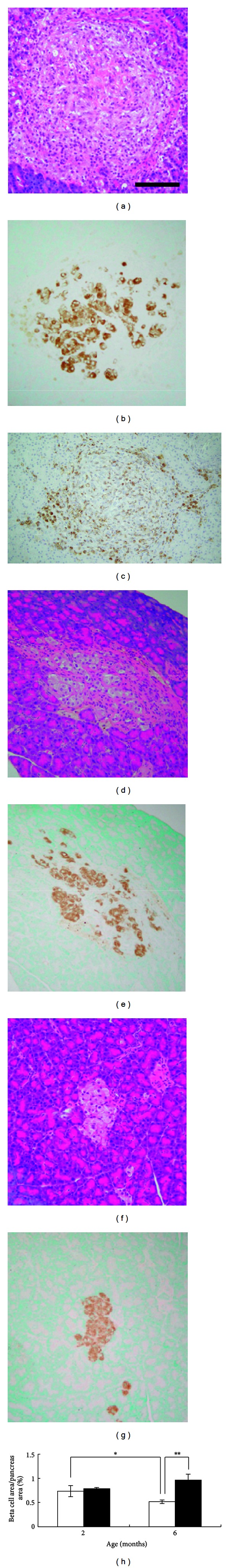
Histopathological appearance of pancreatic islets of male LEA rats. At 2 months of age, H&E staining (a), staining with anti-insulin antibody (b), and staining with anti-ED1 antibody (c). At 12 months of age, H&E staining (d and f) and staining with anti-insulin antibody (e and g). Sections (b), (e), and (g) correspond to sections of (a), (b), and (f). Scale bar = 100 *μ*m. (h) Changes in the proportions of *β*-cells in the pancreas with age. The areas of the *β*-cells and pancreas were calculated by computer-aided imaging. Open and closed bars indicate male LEA and Wistar rats, respectively, at 2 months and 6 months of age. Each value is expressed as the mean ± SD. *P < 0.05. **P < 0.01.

 The age-dependent histological changes in the pancreas were examined (Figure 4). Male LEA rats at 2 months of age had inflammatory reaction in fraction of the pancreatic islets (Figure 4(a)), although the most of islets were intact. Immunostaining for insulin revealed that insulin-positive cells were irregularly distributed within inflammatory foci (Figure 4(b)). The inflamed islets were infiltrated by cells positive for anti-rat macrophage antibody (Figure 4(c)) but not for antibodies against CD4+, CD8+, or CD45RA (data not shown). Fibrosis was also seen in and around large islets at this age. The number and size of islets decreased significantly with age. However, inflammatory reactions disappeared by 6 months of age, and the islets had been replaced by fibrotic remnants in rats over 12 months of age (Figure 4(d)). The number of *β*-cells was reduced, although *β*-cells were present at 12 months of age (Figure 4(e)). A few tiny islets without fibrosis, which appeared to be regenerative islets, were intermingled in the affected pancreas in rats from 12 months of age (Figures 4(f) and 4(g)). The pancreatic islets from female LEA rats had no pathological changes, including fibrosis and inflammatory reaction.

 The volume of *β*-cells relative to the gross volume of the pancreas was determined using the WinROOF software to analyse sections that were immunostained for insulin (Figure 4(h)). In male LEA rats, the volume of *β*-cells significantly decreased from 0.74 ± 0.23% at 2 months to 0.52 ± 0.08% at 6 months. The control Wistar rat strain showed a rising, albeit not statistically significant, trend from 0.79 ± 0.05% at 2 months to 0.96 ± 0.24% at 6 months. These results reveal that the significant decrease in the insulin-positive area with age appears in LEA rats owing to severe fibrosis of the islets. 

To identify whether the diminished *β*-cell mass in LEA rats was caused by a reduction in cell number or atrophy of the cells, we counted the number of nuclei in the insulin-positive areas of 20 islets in five of each of 6-month-old LEA and Wistar rats. The number of nuclei did not differ significantly between the two strains (117.97 ± 4.13 *μ*m^2^/nuclei versus 128.82 ± 13.35 *μ*m^2^/nuclei for LEA versus Wistar rats, resp.). These results indicate that a reduction in the cell number (not atrophy of *β*-cells) is responsible for the reduction of *β*-cell volume in LEA rats.

### 3.5. Pathological Changes of Renal Tissue in Male LEA Rat

**Figure 5:**
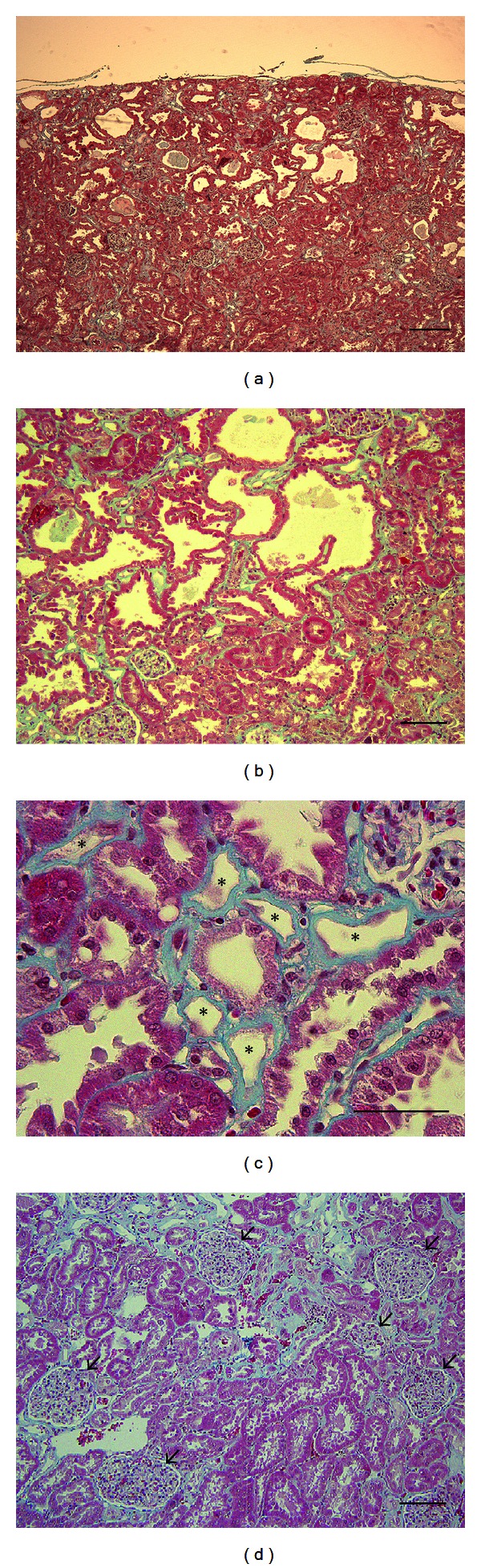
Histopathological appearance of renal tissues from male LEA rats at 12 months of age. Stained with Masson's Trichrome (a–d). Epithelial layer effacement (asterisk), Glomeruli (arrow). Scale bars = 200 *μ*m (a), 100 *μ*m (b and d) and 50 *μ*m (c).

 In LEA rats, glucosuria and proteinuria were present in 100% at 8 months of age and in 66% of males at 12 months of age, respectively. The histopathological analysis was performed to examine the renal lesions in 12-month-old male LEA rats (Figure 5). The large dilatation of tubular lumen was present, mostly in superficial cortex regions (Figure 5(a)). Atrophy of tubular epithelium and flattend/detached renal tubules were also observed (Figure 5(b)). The intracytoplasmic hyaline droplet accumulation and the disappearance of tubular epithelial cell layer associated with thickening of basement membrane were evident in proximal tubules (Figure 5(c)). There were no obvious pathological changes in the glomeruli at 12 months of age (Figure 5(d)). 

## 4. Discussion

 Two inbred strains, Long-Evans Agouti (LEA) and Long-Evans Cinnamon (LEC), which were selected for coat color, were established from a closed colony of Long-Evans rats at the Center for Experimental Plants and Animals, Hokkaido University (Japan) [17]. The LEA rat has been known as the control strain for the LEC rat, an animal model of Wilson disease [18]. However, the large amount of urine and the strong odor of urine, a possible indicator of diabetes, are often observed in LEA rats during long-term breeding. In 1996, we found three male rats that were positive for glucosuria and hyperglycaemia among littermates from an inbred colony of LEA/Hkm. The LEA rats exhibit several distinctive diabetes-related characteristics: (1) onset of diabetes is observed only in male rats, not in female, at over 6 months of age; (2) the early-phase insulin secretion is impaired at 2 months of age; (3) the progressive fibrosis in islet in an age-dependent manner; (4) a normal glycaemic response to exogenous insulin; (5) nonobese. From these results, we conclude that the LEA rat is a new rat model for nonobese type 2 diabetes mellitus.

 Several rat model strains of spontaneous type 2 diabetes mellitus have been identified to date, and they are classified into two types, obesity and nonobesity models. The obesity models of type 2 diabetes, such as Sand [5], Wistar fatty [6], and OLETF rats [7], are characterised by hyperglycaemia, hyperinsulinemia, and insulin resistance. In contrast, the nonobesity models, such as GK [8], WBN/Kob [10], and Spontaneously Diabetic Torii (SDT) rats [11, 19], are characterised by hyperglycaemia, hypoinsulinemia, and noninsulin resistance. We classify the LEA rat as a nonobesity model because BMI of LEA rats is not different from that of control rats. However, there are several differences between the GK and LEA rats. In GK rats, there is no sex difference with respect to the occurrence of diabetes and no age-dependent deterioration of impaired glucose tolerance; in addition, hyperglycaemia occurs 8 days after birth [8, 20]. The SDT rat is a new model of nonobese, severe type 2 diabetes mellitus with hyperglycaemia, hemorrhage in and around the islets, and hyposecretion of insulin (hypoinsulinemia) resulting from a significantly decreased number and size of islets [19]. Although LEA rats displayed no hemorrhage in and around the islets, macrophage infiltration was present around the islets, leading to the progressive fibrosis of islet (Figure 4).

 The glucose intolerance in correspondence with the impairment of the insulin secretion was observed in male LEA (Figure 2 and Table 2), suggesting that the main cause of diabetes in LEA rats is hypoinsulinemia attributable to a decreased number of *β*-cells in the islets (Figure 4). It is also likely that significant decreased capability of early-phase insulin secretion is caused by the hypofunction of *β*-cells. Although there was inflammatory reaction in fraction of the pancreatic islet in male LEA rats at 2 months of age, the volume of *β*-cells in LEA rats was comparable to that of control rats (Figure 4(h)), suggesting that LEA rats have congenital defect of insulin secretion in addition to the progressive reduction of *β*-cell mass in age-dependent manner. The ability to secrete insulin in the early phase reflects the first phase of insulin secretion from pancreatic *β*-cells and contributes to the suppression of gluconeogenesis in the liver [21]. Therefore, we suggest that the LEA rat is unable to suppress the increasing blood glucose concentration that occurs after feeding because of a decreased ability to secrete insulin in the early phase, which eventually leads to the deterioration of *β*-cell function by glucose toxicity and causes the rats to experience chronic hyperglycaemia. The reduction in the number of *β*-cells is thought to be the main cause of type 2 diabetes in LEA rats, and this is supported by previous observations in human type 2 diabetes patients and rat models of type 2 diabetes mellitus [22–26]. 

 Both congenital and acquired factors are involved in the mechanism of *β*-cell reduction. In regard to congenital factors, mutations in transcription factor genes, such as insulin promoter factor-1 (*IPF-1*) [27] and hepatocyte nuclear factor-1*α* (*HNF-1*α**) [28], have been verified. Hyperglycaemia and hyperlipidaemia have been reported as acquired factors. Butler et al. [26] have revealed that apoptosis reduces *β*-cells in human diabetes and that it progresses by amyloid deposition and hyperglycaemia. Zhu et al. [25] have reported that the impairment of *β*-cell proliferation causes a decrease in *β*-cells under hyperglycaemic conditions in OLETF rats, and Movassat et al. [29] have observed that glucose toxicity leads to a reduction in *β*-cells in GK rats. Free-fatty-acid- (FFA-) induced *β*-cell apoptosis has been proposed by Shimabukuro et al. [30] as the underlying cause in Zucker Diabetic Fatty (ZDF) rats. Although it is speculated that the impairment of *β*-cell proliferation, progression of apoptosis by hyperglycaemia, and impairment by cytokines produced by macrophages cause the reduction in *β*-cells in LEA rats, further analyses are required to clarify the pathogenesis of diabetes in LEA rats.

 The systemic complications associated with diabetes are major causes of morbidity and mortality. The abnormality of the renal tubules at 12 months of age was observed in the LEA rats with glycosuria and proteinuria (Figure 5 and Table 1). Onset of diabetes as determined by OGTT was observed in only male LEA rats (Figure 1(a)), and its incidence increased with age: 10% and 61% at 6 and 12 months of age, respectively. However, glucosuria appeared at 5 months of age before the onset of diabetes in male rats, and was present in 100% of the females at 9 months (Table 1), which did not develop diabetes (Figure 1(a)). Based on these findings, it is unlikely that the onset of diabetes and impairment of glucose intolerance are not associated with glucosuria and proteinuria. Although the LEA rat is used as the control strain of the LEC rat since they do not harbor *Atp7b *mutation [31], the several phenotypes such as hypersensitivity to X-rays [32] and the lack of D-amino acid oxidase (DAO) activity [33], which is involved in the degradation of D-serine, a key coagonist for N-methyl-d-aspartate (NMDA) receptor, have been reported. We are now performing quantitative trait locus (QTL) analyses for impaired glucose tolerance and urinary glucose to lead us to identification of genes for glucose intolerance, renal glucose excretion, and the development of diabetes in LEA rat.

 In conclusion, the LEA rat has distinctive characteristics that are different from the previously described model rats. The LEA rats develop late onset diabetes in correspondence with the impairment of the insulin secretion, which is caused by progressive fibrosis in pancreatic islets in age-dependent manner. In Japan, the prevalence of type 2 diabetes mellitus is increasing rapidly, and more than 10% of individuals over 40 years of age are affected. Relatively few diabetic individuals in Japan are obese, and impairment of insulin secretion often develops before onset of diabetes [34]. The unique characteristics of LEA rat are a great advantage to analyze the progression of diabetes mellitus with age. The additional studies are expected to disclose the genes involved in type 2 diabetes mellitus.

## Supplementary Material

The supplementary Figure 1 shows the survival rate of LEA rats.

## Figures and Tables

**Table 1:** Incidence of glucosuria and proteinuria in LEA rats (%).

Condition	Sex	Age (months)							
		5	6	7	8	9	10	11	12
Glucosuria	Male	13	50	88	100	100			
	Female	0	0	25	88	100			
Proteinuria	Male	0	14	21	43	57	48	52	66
	Female	0	0	0	0	20	20	15	30

*n* = 32 for males; *n* = 35 for females.

**Table 2:** Insulinogenic index (ΔI.I.) for primary insulin secretion in male LEA rat.

Strain	Age (months)	
	2	12
LEA	0.103 ± 0.07*	0.153 ± 0.04*
Wistar	0.608 ± 0.191	0.583± 0.269

*P < 0.05 compared to age matched Wistar rat.

## References

[1] Zimmet P, Alberti KGMM, Shaw J (2001). Global and societal implications of the diabetes epidemic. *Nature*.

[2] Alberti KG, Zimmet PZ (1998). Definition, diagnosis and classification of diabetes mellitus and its complications. Part 1: diagnosis and classification of diabetes mellitus provisional report of a WHO consultation. *Diabetic Medicine*.

[3] King H, Aubert RE, Herman WH (1998). Global burden of diabetes, 1995–2025: prevalence, numerical estimates, and projections. *Diabetes Care*.

[4] Zimmet P (2000). Globalization, coca-colonization and the chronic disease epidemic: can the doomsday scenario be averted?. *Journal of Internal Medicine*.

[5] Hackel DB, Mikat E, Lebovitz HE, Schmidt-Nielsen K, Horton ES, Kinney TD (1967). The sand rat (psammomys obesus) as an experimental animal in studies of diabetes mellitus. *Diabetologia*.

[6] Ikeda H, Shino A, Matsuo T, Iwatsuka H, Suzuoki Z (1981). A new genetically obese-hyperglycemic rat (Wistar fatty). *Diabetes*.

[7] Kawano K, Hirashima T, Mori S, Saitoh Y, Kurosumi M, Natori T (1992). Spontaneous long-term hyperglycemic rat with diabetic complications: Otsuka Long-Evans Tokushima Fatty (OLETF) strain. *Diabetes*.

[8] Goto Y, Kakizaki M, Masaki N (1975). Spontaneous diabetes produced by selective breeding of normal Wistar rats. *Proceedings of the Japan Academy*.

[9] Martinez SM, Tarres MC, Montenegro S (1988). Spontaneous diabetes in eSS rats. *Acta Diabetologica Latina*.

[10] Nakama K, Shichinohe K, Kobayashi K (1985). Spontaneous diabetes-like syndrome in WBN/Kob rats. *Acta Diabetologica Latina*.

[11] Shinohara M, Masuyama T, Shoda T (2000). A new spontaneously diabetic non-obese torii rat strain with severe ocular complications. *International Journal of Experimental Diabetes Research*.

[12] Gauguier D, Froguel P, Parent V (1996). Chromosomal mapping of genetic loci associated with non-insulin dependent diabetes in the GK rat. *Nature Genetics*.

[13] Wei S, Wei K, Moralejo DH (1999). Mapping and characterization of quantitative trait loci for non-insulin- dependent diabetes mellitus with an improved genetic map in the Otsuka Long-Evans Tokushima Fatty rat. *Mammalian Genome*.

[14] Masuyama T, Fuse M, Yokoi N (2003). Genetic analysis for diabetes in a new rat model of nonobese type 2 diabetes, Spontaneously Diabetic Torii rat. *Biochemical and Biophysical Research Communications*.

[15] Sasaki M, Yoshida MC, Kagami K (1985). Spontaneous hepatitis in an inbred strain of Long-Evans rats. *Rat News Letter*.

[16] Bouwens L, Wang RN, de Blay E, Pipeleers DG, Kloppel G (1994). Cytokeratins as markers of ductal cell differentiation and islet neogenesis in the neonatal rat pancreas. *Diabetes*.

[17] Kasai N, Osanai T, Miyoshi I, Kamimura E, Yoshida MC, Dempo K (1990). Clinico-pathological studies of LEC rats with hereditary hepatitis and hepatoma in the acute phase of hepatitis. *Laboratory Animal Science*.

[18] Li Y, Togashi Y, Sato S (1991). Spontaneous hepatic copper accumulation in Long-Evans cinnamon rats with hereditary hepatitis: a model of Wilson’s disease. *Journal of Clinical Investigation*.

[19] Masuyama T, Komeda K, Hara A (2004). Chronological characterization of diabetes development in male Spontaneously Diabetic Torii rats. *Biochemical and Biophysical Research Communications*.

[20] Yagihashi S, Goto Y, Kakizaki M, Kaseda N (1978). Thickening of glomerular basement membrane in spontaneously diabetic rats. *Diabetologia*.

[21] Williams G, Pickup JC (2004). *Handbook of Diabetes*.

[22] Saito K, Yaginuma N, Takahashi T (1979). Differential volumetry of A, B and D cells in the pancreatic islets of diabetic and nondiabetic subjects. *Tohoku Journal of Experimental Medicine*.

[23] Movassat J, Saulnier C, Portha B (1995). *β*-cell mass depletion precedes the onset of hyperglycaemia in the GK rat, a genetic model of non-insulin-dependent diabetes mellitus. *Diabete et Metabolisme*.

[24] Pick A, Clark J, Kubstrup C (1998). Role of apoptosis in failure of *β*-cell mass compensation for insulin resistance and *β*-cell defects in the male Zucker diabetic fatty rat. *Diabetes*.

[25] Zhu M, Noma Y, Mizuno A, Sano T, Shima K (1996). Poor capacity for proliferation of pancreatic *β*-cells in Otsuka-Long- Evan-Tokushima fatty rat: a model of spontaneous NIDDM. *Diabetes*.

[26] Butler AE, Janson J, Bonner-Weir S, Ritzel R, Rizza RA, Butler PC (2003). *β*-cell deficit and increased *β*-cell apoptosis in humans with type 2 diabetes. *Diabetes*.

[27] Clocquet AR, Egan JM, Stoffers DA (2000). Impaired insulin secretion and increased insulin sensitivity in familial maturity-onset diabetes of the young 4 (insulin promoter factor 1 gene). *Diabetes*.

[28] Pontoglio M, Sreenan S, Roe M (1998). Defective insulin secretion in hepatocyte nuclear factor 1*α*-deficient mice. *Journal of Clinical Investigation*.

[29] Movassat J, Saulnier C, Serradas P, Portha B (1997). Impaired development of pancreatic beta-cell mass is a primary event during the progression to diabetes in the GK rat. *Diabetologia*.

[30] Shimabukuro M, Zhou YT, Levi M, Unger RH (1998). Fatty acid-induced *β* cell apoptosis: a link between obesity and diabetes. *Proceedings of the National Academy of Sciences of the United States of America*.

[31] Wu J, Forbes JR, Chen HS, Cox DW (1994). The LEC rat has a deletion in the copper transporting ATPase gene homologous to the Wilson disease gene. *Nature Genetics*.

[32] Masuda K, Miyamoto T, Cho AR, Agui T (2006). Analysis of the cell cycle of fibroblasts derived from the LEC rat after X-irradiation. *Japanese Journal of Veterinary Research*.

[33] Konno R, Okamura T, Kasai N, Summer KH, Niwa A (2009). Mutant rat strain lacking D-amino-acid oxidase. *Amino Acids*.

[34] Fukushima M, Suzuki H, Seino Y (2004). Insulin secretion capacity in the development from normal glucose tolerance to type 2 diabetes. *Diabetes Research and Clinical Practice*.

