# Supplementary figures and images for: Phenotypic Characterization of LEA Rat: A New Rat Model of Nonobese Type 2 Diabetes
Source: J Diabetes Res. 2013 Feb 26;2013:986462. doi: 10.1155/2013/986462 (PMC3647576; doi:10.1155/2013/986462)

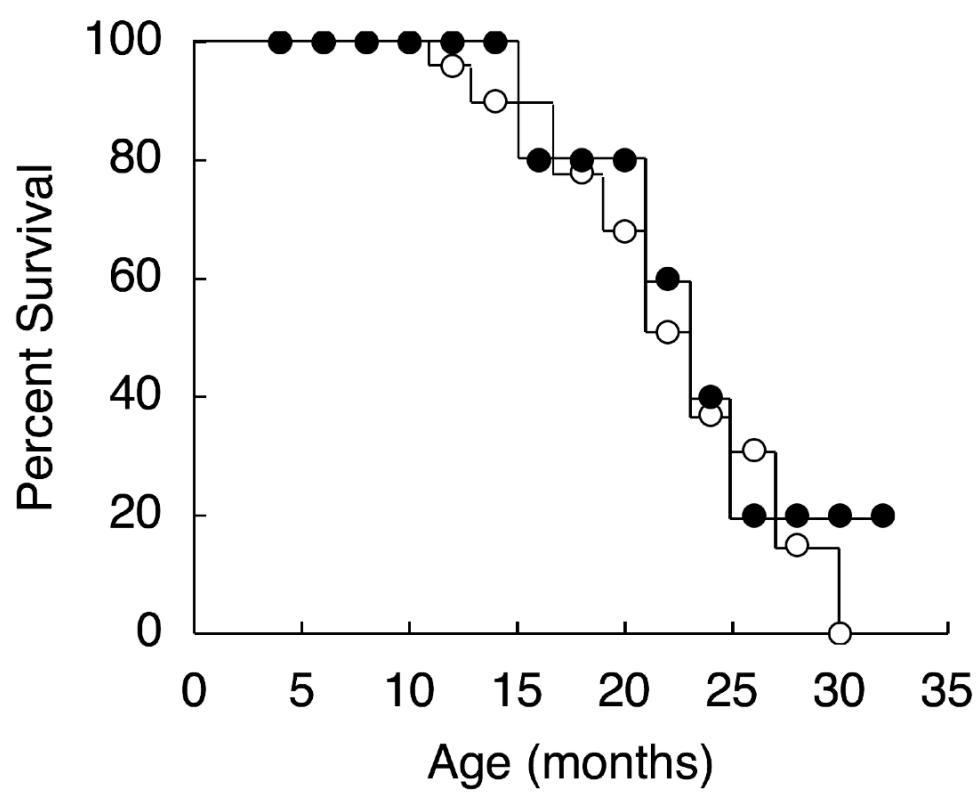

Supplementary Figure 1

Supplement: Supplementary file 1 — The supplementary Figure 1 shows the survival rate of LEA rats. [file 986462.f1.pdf]
